# Risk factors for disease severity in hypertriglyceridemic acute pancreatitis: a single-center retrospective study

**DOI:** 10.1186/s12876-026-04608-9

**Published:** 2026-01-13

**Authors:** Rong-Rong Wei, Yan Zhou, Haifeng Yuan, Lele Zong, Chengchao Gao, Donglin Yan, Dongmei Guo

**Affiliations:** Heze Municipal Hospital, Heze, Shandong China

**Keywords:** Hypertriglyceridemic acute pancreatitis, Risk factor, Triglyceride, Serum calcium, C-reactive protein, Apolipoprotein A1

## Abstract

**Background:**

Hypertriglyceridemia is an increasingly common cause of acute pancreatitis (AP). Patients with hypertriglyceridemic acute pancreatitis (HTG-AP) have higher complication and mortality rates compared to those with other etiologies. Early prediction of disease severity remains challenging due to the lack of readily available tools specifically for HTG-AP.

**Method:**

This was a single-center retrospective cohort study. A total of 214 HTG-AP patients were classified into mild acute pancreatitis (MAP, *n* = 106) and moderately severe/severe acute pancreatitis (MSAP/SAP, *n* = 108) groups based on the revised Atlanta criteria. Clinical characteristics and laboratory parameters were compared between the two groups. Binary logistic regression analysis and ROC analysis were performed to identify risk factors and develop a combined predictive model. Bootstrap analysis was performed for internal validation, and calibration curves were utilized to evaluate model calibration.

**Results:**

The MSAP/SAP group exhibited elevated triglyceride (TG), amylase (AMY), blood glucose (GLU), C-reactive protein (CRP), and white blood cell (WBC) levels, but lower serum calcium (Ca^2+^) and apolipoprotein A1 (ApoA1) levels. Binary logistic regression analysis identified several independent risk factors for MSAP/SAP: TG, CRP, WBC, and Ca^2+^. The combined predictive model achieved an area under the curve (AUC) of 0.837. At the optimal cut-off value of 0.48, the sensitivity and specificity of the combined predictive indicator were 77.4% and 75.0%, respectively. Bootstrap validation demonstrated that the 95% confidence intervals for the regression coefficients of TG, Ca^2+^, CRP, and WBC did not include zero. The Hosmer–Lemeshow goodness-of-fit test showed a *p*-value of 0.312 (>0.05).

**Conclusion:**

Elevated TG, CRP, and WBC levels, as well as decreased Ca^2+^, are independent risk factors for severe HTG-AP. A combined model based on these readily available early parameters demonstrates robust predictive performance, stability, and calibration.

## Introduction

With the improvement of people’s living standards and dietary changes in China, hyperlipidemia prevalence is rising. Hyperlipidemia has become increasingly recognized as one of the common causes of acute pancreatitis (AP), following biliary and alcoholic etiologies. Studies have shown that hypertriglyceridemia aggravates the severity of AP and increases the risk of local and systemic complications. Hypertriglyceridemic acute pancreatitis (HTG-AP) is associated with worse clinical outcomes, including higher rates of organ failure, shock, systemic inflammatory response syndrome, and mortality [1, 2]. Therefore, it is important to accurately assess and predict HTG-AP severity at an early stage.

Although markers such as serum calcium and C-reactive protein [3], along with composite scores including the Ranson score, Acute Physiology and Chronic Health Evaluation II (APACHE II), and Modified CT Severity Index (MCTSI) [4], are used to assess severity in general AP, they have not been specifically validated for HTG-AP. Moreover, the predictive value of single biological indicators for HTG-AP is limited. The Ranson score, APACHE II score, MCTSI, and other scoring systems often require multiple complex parameters. Some of these parameters are not readily available in the early phase, which limits their utility for rapid bedside risk assessment at initial presentation. This creates a clear clinical gap: there is no specific predictive model that integrates readily available early parameters for immediate use in HTG-AP patients.

To address this gap, this study aims to identify early risk factors for disease severity in HTG-AP and to develop a combined predictive model based on routinely available parameters. This approach is intended to facilitate early identification of high-risk patients and enable timely intervention.

## Materials and methods

This was a single-center retrospective study that included all patients diagnosed with HTG-AP at Heze Municipal Hospital between April 2018 and December 2024. A total of 214 hospitalized patients with HTG-AP were enrolled. The patients were divided into a mild acute pancreatitis (MAP) group (*n* = 106) and a moderately severe/severe acute pancreatitis (MSAP/SAP) group (*n* = 108). Clinical characteristics and laboratory parameters were compared between the two groups. Binary logistic regression analysis was used to identify risk factors associated with disease severity in HTG-AP. Receiver operating characteristic (ROC) curve analysis was performed to evaluate the predictive performance of the combined indicators. Internal validation of the predictive model was conducted using bootstrap resampling with 1,000 repetitions. Model calibration was assessed using the Hosmer–Lemeshow goodness-of-fit test and calibration curve analysis.

### Study population

This single-center retrospective cohort study initially screened all inpatients with a primary diagnosis of AP at Heze Municipal Hospital between April 1, 2018, and December 31, 2024. A total of 1,096 patients with AP were identified from the electronic medical record system. Patients were subsequently screened according to the following steps: (1) exclusion of 2 patients younger than 18 years; (2) exclusion of 11 pregnant patients; (3) exclusion of 137 patients with recurrent pancreatitis; (4) exclusion of 598 patients with other etiologies, including 422 cases of biliary pancreatitis, 167 cases of alcoholic pancreatitis, 7 cases of traumatic or postoperative pancreatitis, 1 case of drug-induced pancreatitis, and 1 case of tumor-related pancreatitis. (5) For patients suspected of hypertriglyceridemic etiology, the following diagnostic criteria were applied: (i) serum triglyceride (TG) level ≥ 11.3 mmol/L; or (ii) TG level between 5.65 and 11.3 mmol/L with concomitant chylous serum. At this stage, 125 patients who did not meet the TG criteria were excluded. (6) Nine patients with incomplete clinical data that precluded severity classification were excluded.

Ultimately, 214 patients with HTG-AP who met all inclusion criteria were enrolled in the final analysis cohort.

### Criteria

- Inclusion criteria:
  - ① Age >18 years.
  - ② Diagnosis meeting the revised Atlanta criteria for AP [5]：with serum TG level ≥11.3 mmol/L, or TG level between 5.65 and 11.3 mmol/L with chylous serum.
  - ③ First episode of HTG-AP.
  - ④ Complete clinical data and medical records available.
- Exclusion criteria:
  - ① Pregnancy.
  - ② Pancreatitis due to other causes, including biliary disease, alcohol, trauma, tumors, or complications from surgery or examination.
  - ③ History of chronic pancreatitis or recurrent pancreatitis.
  - ④Missing critical clinical data affecting accurate severity classification.

### Observation indicators

Data on patient characteristics, laboratory parameters, and imaging studies were collected from the hospital database. General patient characteristics included sex, age, hospital stay duration, comorbidities, treatment methods, clinical outcomes, and medical expenses. Laboratory parameters included white blood cell (WBC), C-reactive protein (CRP), amylase (AMY), TG, blood glucose (GLU), apolipoprotein A1 (ApoA1), serum calcium (Ca^2+^), urea nitrogen (UREA), creatinine (Cre), alanine aminotransferase (ALT), aspartate aminotransferase (AST), alkaline phosphatase (ALP), γ-glutamyltransferase (GGT), and total bilirubin (TBIL). All laboratory parameters were measured from the initial blood sample obtained before initiating any specific therapy for HTG-AP (e.g., insulin infusion or plasmapheresis). Due to the urgency of patients’ conditions, TG levels at admission were not always obtained under strictly fasting conditions. The reference range for AMY was 0–180 U/L. Imaging studies included abdominal CT or MRI. Timing of imaging was based on diagnostic certainty at admission. For patients with uncertain diagnoses based on symptoms and serum enzyme levels, CT or MRI was typically performed within 72 h after admission to confirm diagnosis. For patients with a clear clinical and biochemical diagnosis, imaging was performed after 72 h.

### Grouping

Due to the small sample size of the SAP group (*n* = 11), we combined the MSAP and SAP groups into one group (MSAP/SAP) to enhance statistical power. This approach is consistent with methodologies employed in previous studies. Patients were divided into two groups: MAP and MSAP/SAP. Disease severity was classified based on the revised Atlanta classification for AP [5]:① MAP: patients exhibiting changes in laboratory indicators and clinical manifestations without local or systemic complications or organ failure. ② MSAP: patients with laboratory and clinical manifestations accompanied by transient organ failure (resolved within 48 h) or local or systemic complications. ③ SAP: patients with laboratory and clinical manifestations accompanied by persistent organ failure (lasting > 48 h). The modified Marshall scoring system was used to assess organ failure.

### Statistical methods

All statistical analyses were performed using SPSS software (version 27 for Windows). Continuous variables were presented as mean ± standard deviation or median (interquartile range [IQR]) based on normality testing. Categorical variables were presented as numbers (percentages). Differences between groups were analyzed using the independent-samples t-test, Mann–Whitney U test, or chi-square test, as appropriate. Risk factors for disease severity were identified by binary logistic regression analysis. The predictive performance of the indicators was evaluated using ROC curves, calculating the area under the curve (AUC), sensitivity, and specificity. Internal validation of the model was conducted by bootstrap resampling (1,000 repetitions). Model calibration was assessed using the Hosmer–Lemeshow goodness-of-fit test and calibration curves. *P* < 0.05 was considered statistically significant.

## Result

### Clinical characteristics

The general characteristics of the patients are presented in Table 1. The median age was 38 years (IQR: 34–46), and the male-to-female ratio was 5.29 (180 males and 34 females). Pre-existing comorbidities (e.g., diabetes, fatty liver, hypertension) were present in 57.5% of the patients. Compared to the MAP group, the MSAP/SAP group had a significantly longer hospital stay (*P* < 0.001), higher modified Marshall scores (*P* < 0.001), and higher MCTSI scores (*P* < 0.001). Patients with more severe HTG-AP had higher utilization rates of blood purification therapy (22.2%, *P* < 0.001). Two patients died during hospitalization. There was no significant difference in the recurrence rate between the MSAP/SAP and MAP groups (*P* = 0.592). Rates of invasive interventions and ICU admission were significantly higher in the MSAP/SAP group. Specifically, patients in the MSAP/SAP group underwent gastrointestinal decompression (24.1%, *P* < 0.001), received enteral nutritional support (10.2%, *P* = 0.001), and required ICU admission (28.7% vs. 0.0%, *P* < 0.001) more frequently.

**Table 1:** Clinical characteristics of patients in the MSAP/SAP and MAP groups

Characteristics	Total (*n* = 214)	MAP (*n* = 106)	MSAP + SAP (*n* = 108)	*P* value
Gender, *n* (%)				0.07
Male	180 (84.1)	94 (88.7)	86 (79.6)	
Female	34 (15.9)	12 (11.3)	22 (20.4)	
Age (year)	38 (34, 46)	39 (34, 46)	37 (34, 42)	0.103
Length of hospital stay (day)	8 (6,11.25)	7 (5,8)	11 (8,13)	<0.001
Comorbidities, *n* (%)	123 (57.5)	68 (64.2)	55 (50.9)	0.05
Diabetes	71 (33.2)	37 (34.9)	34 (31.5)	0.595
Fatty liver	156 (72.9)	74 (69.8)	82 (75.9)	0.314
Hypertension	42 (19.6)	22 (20.8)	20 (18.5)	0.68
Cerebral infarction	2 (0.9)	1 (0.9)	1 (0.9)	1
Coronary atherosclerotic heart disease	3 (1.4)	2 (1.9)	1 (0.9)	0.987
Gout	2 (0.9)	1 (0.9)	1 (0.9)	1
Modified Marshall scores	0 (0, 0)	0 (0, 0)	0 (0, 1)	<0.001
MCTSI scores	4 (2, 4)	2 (2, 2)	4 (4, 6)	<0.001
Treatment modalities, *n* (%)				
Blood purification therapy	24 (11.2)	0 (0.0)	24 (22.2)	<0.001
Invasive interventions, *n* (%)				
Peritoneal fluid drainage	2 (0.9)	0 (0.0)	2 (1.9)	0.498
Gastrointestinal decompression	32 (15.0)	6 (5.7)	26 (24.1)	<0.001
Enteral nutrition	11 (5.1)	0 (0.0)	11 (10.2)	0.001
Surgical intervention	0 (0.0)	0 (0.0)	0 (0.0)	-
Intensive Care Unit (ICU) admission	31 (14.5)	0 (0.0)	31 (28.7)	<0.001
Clinical outcomes, *n* (%)				0.498
Survival	212 (99.1)	106 (100.0)	106 (98.1)	
Death	2 (0.9)	0 (0.0)	2 (1.9)	
Recurrence rate, *n* (%)	65 (30.4)	34 (32.1)	31 (28.7)	0.592
TG (mmol/l)	14.28 (12.04, 16.6)	13.61 (11.59, 15.44)	14.61 (13.04, 21.65)	0.009
AMY (U/L)	170.5 (84.75, 331.5)	151.5 (76.5, 296.75)	172.5 (91.25, 336.25)	0.026
Ca^2+^(mmol/l)	2.19 (2.02, 2.33)	2.25 (2.08, 2.35)	2.09 ± 0.31	0.002
Glu (mmol/l)	11.38 (7.92, 15.15)	11.11 (7.13, 13.95)	11.59 (8.65, 16.37)	0.028
CRP (mg/l)	96.45 (31.15, 191.96)	54.4 (13.93, 137.23)	129.93 (64.8, 241.73)	<0.001
WBC (×10^9^/L)	12 ± 4.52	10.77 ± 3.36	14.47 ± 4.78	<0.001
ALT (U/L)	17 (11.75, 28)	18.5 (13, 32)	17 (10, 25)	0.06
AST (U/L)	18 (13, 25)	18 (13, 24.25)	18 (13, 26)	0.936
GGT (U/L)	55 (33, 103)	54 (33, 114.25)	56 (32.25, 90.5)	0.415
ALP (U/L)	77 (65, 91)	79.5 (67, 92)	76 (64, 89.75)	0.47
TBIL (umol/L)	12.5 (8.58, 17)	12.5 (8.5, 17)	12.6 (8.63, 17.08)	0.974
Cre (umol/l)	61.5 (52, 74)	62 (52, 73.48)	61 (51.25, 78)	0.692
UREA (umol/L)	4.1 (3.2, 5.39)	4 (3.2, 5.29)	4.25 (3.2, 5.6)	0.519
ApoA1 (g/L)	0.99 ± 0.39	1.07 ± 0.34	0.92 ± 0.42	0.005

Furthermore, TG (*P* = 0.009), AMY (*P* = 0.026), GLU (*P* = 0.028), CRP (*P* < 0.001), and WBC (*P* < 0.001) levels were significantly higher in the MSAP/SAP group. In contrast, Ca^2+^ (*P* = 0.002) and ApoA1 (*P* = 0.005) levels were significantly lower in this group. No significant differences were observed in AST, ALT, GGT, ALP, TBIL, Cre, or UREA levels between the two groups (*P* > 0.05).

Complications in the MSAP/SAP group are presented in Table 2. Among the 108 patients, the incidence rates of local and systemic complications were 38.9% and 69.4%, respectively. Systemic inflammatory response syndrome (SIRS) was the most common complication, occurring in 66.7% of the MSAP/SAP cohort. Compared to the MSAP subgroup (*n* = 97), the SAP subgroup had higher incidences of severe complications, such as pancreatic necrosis, acute renal failure (ARF), acute respiratory distress syndrome (ARDS), and sepsis.

**Table 2:** Complications in MSAP and SAP groups

Complications, *n* (%)	Total (*n* = 108)	MSAP (*n* = 97)	SAP (*n* = 11)
Local complications	42 (38.9)	33 (34.0)	9 (81.8)
Pancreatic Necrosis	12 (11.1)	4 (4.1)	8 (72.7)
Acute Peripancreatic Fluid Collection (APFC)	39 (36.1)	30 (30.9)	9 (81.8)
Systemic complications	75 (69.4)	64 (66.0)	11 (100)
Systemic Inflammatory Responsesyndrome (SIRS)	72 (66.7)	63 (64.9)	9 (81.8)
Acute Renal Failure (ARF)	5 (4.6)	1 (1.0)	4 (36.4)
Acute Respiratory Distress Syndrome (ARDS)	6 (5.6)	0 (0.0)	6 (54.5)
Shock	2 (1.9)	0 (0.0)	2 (18.2)
Sepsis	3 (2.8)	0 (0.0)	3 (27.3)
Intra-Abdominal Hypertension (IAH)	2 (1.9)	0 (0.0)	2 (18.2)

Diagnostic criteria for enrolled HTG-AP patients are shown in Table 3. All 214 patients met the revised Atlanta criteria for AP. A total of 31 patients (14.5%) were diagnosed based on typical abdominal pain and serum amylase levels ≥ 3 times the upper normal limit. The remaining 183 patients (85.5%) had amylase levels below this threshold at admission and were diagnosed based on typical abdominal pain and imaging findings (CT or MRI).

**Table 3:** Diagnostic criteria of enrolled HTG-AP patients

Diagnostic criteria	*n* (%)
Abdominal pain and characteristic imaging	183 (85.5)
Abdominal pain and elevated serum enzymes	31 (14.5)

### Binary logistic regression analysis

The results of univariate binary logistic regression analysis are presented in Table 4. To identify factors associated with disease severity, binary logistic regression analyses were performed. The analysis showed that elevated TG (*P* = 0.005), CRP (*P* = 0.001), and WBC (*P* < 0.001), as well as decreased Ca^2+^ (*P* = 0.013), were significantly associated with MSAP/SAP. AMY, ApoA1, and GLU showed no significant associations (*P* > 0.05).

**Table 4:** Univariate binary logistic regression analysis

Parameter	B	OR	95% CI	*P* value
AMY	0.001	1.001	1.000–1.002	0.083
TG	0.054	1.056	1.017–1.096	0.005
Ca^2+^	−1.787	0.167	0.041–0.689	0.013
CRP	0.005	1.005	1.002–1.009	0.001
WBC	0.293	1.340	1.211–1.483	<0.001
ApoA1	−0.645	0.525	0.186–1.479	0.222
GLU	0.008	1.008	0.935–1.087	0.835

The four significant variables were entered into multivariate logistic regression analysis. As shown in Table 5, TG (B = 0.06, OR = 1.062, *P* = 0.001), Ca^2+^ (B = −2.14, OR = 0.118, *P* = 0.002), CRP (B = 0.006, OR = 1.006, *P* < 0.001), and WBC (B = 0.279, OR = 1.322, *P* < 0.001) were all significant predictors. The analysis identified hypertriglyceridemia, hypocalcemia, elevated CRP, and elevated WBC as key risk factors for MSAP/SAP in patients with HTG-AP.

**Table 5:** Multivariate binary logistic regression analysis

Parameter	B	OR	95% CI	*P* value
TG	0.06	1.062	1.026–1.1	0.001
Ca^2+^	−2.14	0.118	0.03–0.466	0.002
CRP	0.006	1.006	1.003–1.009	<0.001
WBC	0.279	1.322	1.199–1.456	<0.001

### ROC curve analysis

**Fig. 1:**
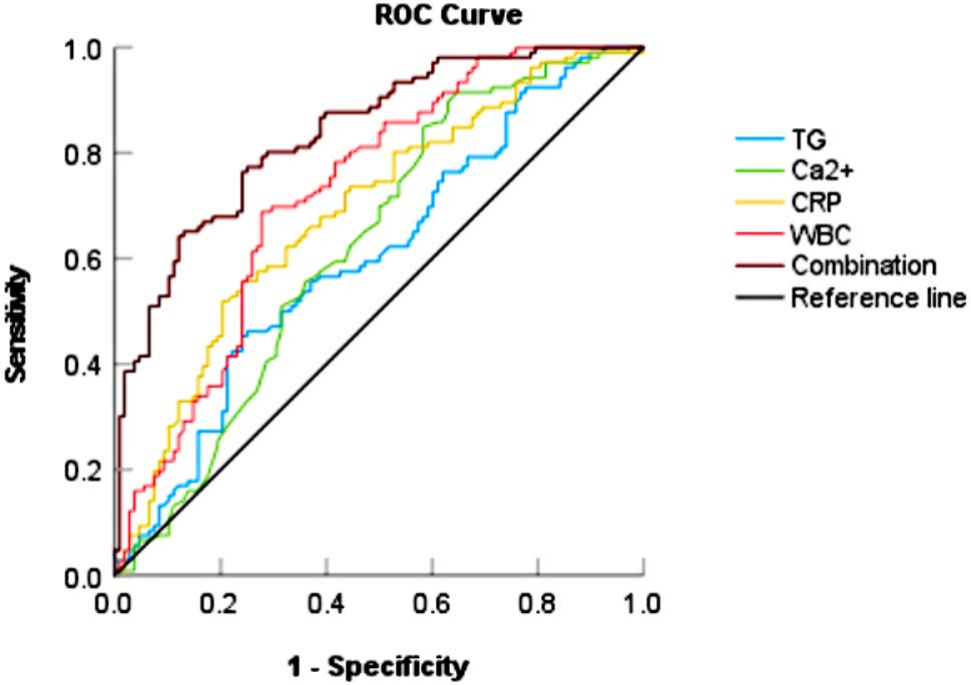
ROC curve of TG, Ca^2+^, CRP, WBC, and their combination for predicting HTG-AP severity

The predictive value of TG, Ca^2+^, WBC, CRP, and their combination for HTG-AP severity is shown in Table 6. A combined predictor was developed using binary logistic regression incorporating these indicators. To evaluate the predictive ability, ROC curves were plotted (Fig. 1). The AUC values for TG (AUC = 0.603, 95% CI: 0.528–0.679, *P* = 0.009), Ca^2+^ (AUC = 0.625, 95% CI: 0.55–0.7, *P* = 0.002), CRP (AUC = 0.688, 95% CI: 0.617–0.759, *P* < 0.001), and WBC (AUC = 0.726, 95% CI: 0.658–0.794, *P* < 0.001) exceeded 0.5, indicating some predictive value for HTG-AP severity. The combined predictor demonstrated significantly superior predictive performance, with an AUC of 0.837. At the optimal cut-off value of 0.48, its sensitivity and specificity were 77.4% and 75%, respectively. These findings suggest that the combined predictor outperforms individual markers.

**Table 6:** Predictive value of TG, Ca^2+^, WBC, CRP, and their combination

Parameter	AUC	95% CI	*P* value	Optimal threshold	Sensitivity (%)	Specificity (%)	Youden Index
TG	0.603	0.528–0.679	0.009	12.97	0.759	0.453	0.212
CRP	0.688	0.617–0.759	<0.001	64.45	0.759	0.557	0.316
WBC	0.726	0.658–0.794	<0.001	12.02	0.722	0.689	0.411
Ca^2+^	0.625	0.55–0.7	0.002	0.50	0.352	0.915	0.267
Combination	0.837	0.785–0.889	<0.001	0.484	0.774	0.75	0.524

### Internal validation and calibration of the prediction model

Internal validation was conducted using bootstrap resampling with 1,000 iterations to assess the stability and calibration of the prediction model. The bootstrap analysis confirmed the stability of the four independent predictors. The 95% confidence intervals for regression coefficients of TG, Ca^2+^, CRP, and WBC, derived from bootstrap samples, did not include zero (Table 7), indicating stable predictor effects.

**Table 7:** Bootstrap analysis evaluating predictor stability in the prediction model

Parameter	B	Bias	Standard Error	*P* value	95%CI
TG	0.06	0.003	0.021	0.002	0.028–0.112
Ca^2+^	−2.14	−0.107	0.811	0.005	−4.073- −0.823
CRP	0.006	0.000	0.002	0.001	0.003–0.009
WBC	0.279	0.011	0.05	0.001	0.201–0.402

**Fig. 2:**
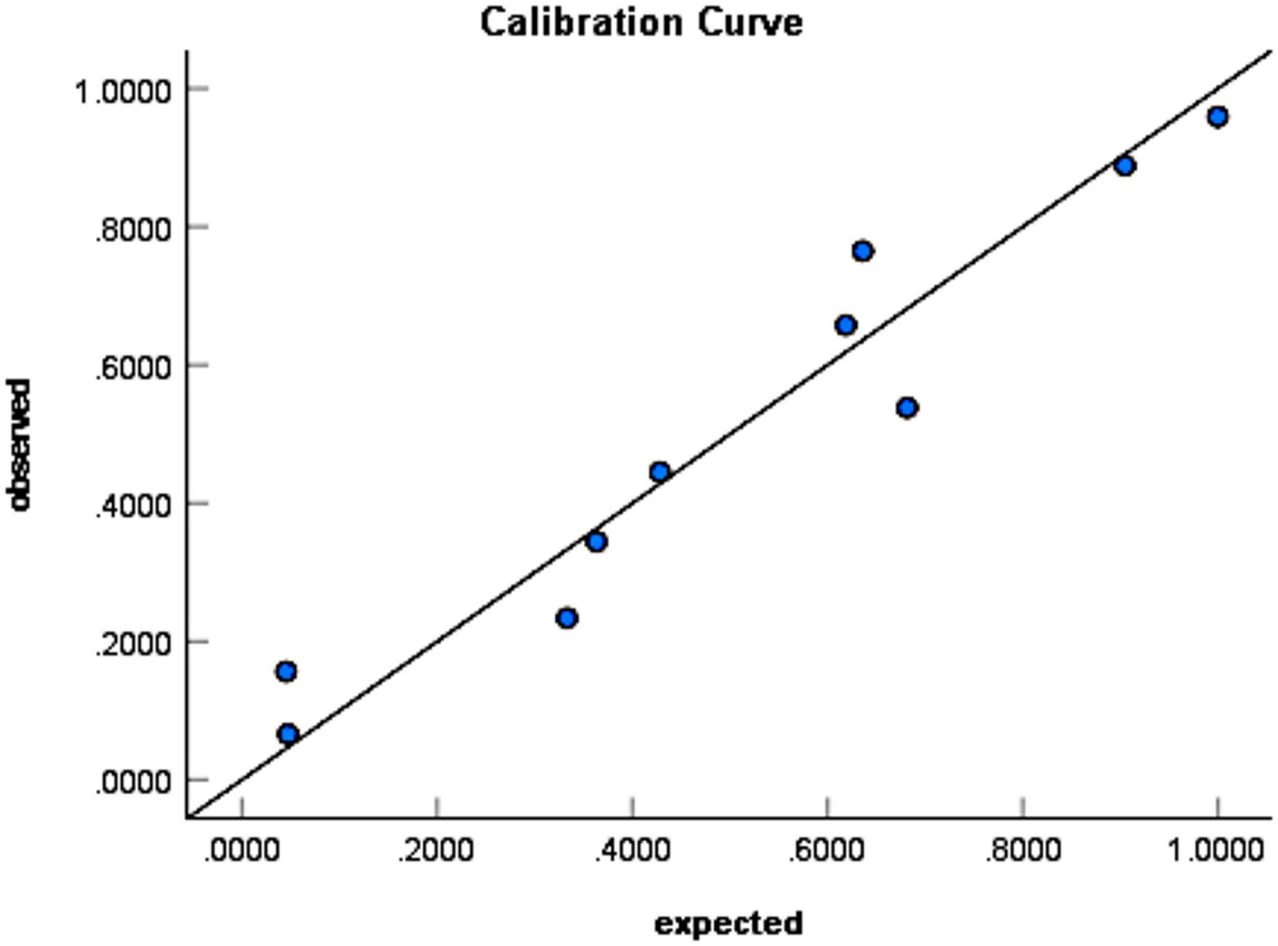
Calibration curve of the prediction model

The Hosmer–Lemeshow goodness-of-fit test was performed to evaluate model calibration (χ² = 9.369, df = 8, *P* = 0.312). The calibration curve is shown in Fig. 2, demonstrating excellent consistency between predicted probabilities and observed outcomes.

## Discussion

This study shows that the median age of HTG-AP patients was 38 years, with a predominantly male incidence. These findings are consistent with previous reports indicating that HTG-AP patients tend to be younger and male [6, 7]. This may be related to higher rates of alcohol consumption and metabolic comorbidities among men [8]. Patients in the MSAP/SAP group had longer hospital stays and higher Marshall and MCTSI scores compared to the MAP group. Significant differences were also observed between groups in TG, AMY, GLU, CRP, and WBC levels. No significant differences in age, pre-existing comorbidities, AST, ALT, GGT, ALP, TBIL, or recurrence rates were found between groups. Additionally, no significant differences in mortality, Cre, or UREA were observed, likely due to the low incidence of patient deaths and ARF in this study. Clinical characteristic analysis indicated a significantly higher utilization rate of blood purification therapy in the MSAP/SAP group. Blood purification therapy can rapidly lower TG levels and effectively remove toxins and inflammatory mediators (e.g., interleukin-1 [IL-1], tumor necrosis factor-alpha [TNF-α]). It also corrects fluid, electrolyte, and acid-base imbalances, thereby maintaining homeostasis. However, blood purification therapy is expensive and complex in operation, and it carries potential risks such as bleeding, infection, and thrombosis. Therefore, Blood purification therapy is recommended only for HTG-AP patients who receive non-invasive treatment after admission but whose serum TG levels remain > 1000 mg/dL (11.3 mmol/L) or fail to decrease by 50% after 24–48 h of treatment [9, 10].

The results showed a statistically significant difference in TG levels among patients with different disease severities (*P* = 0.009). Binary logistic regression analysis identified elevated TG as an independent risk factor for MSAP/SAP (*P* = 0.004, OR = 1.058). This finding is consistent with previous animal studies, which indicated that higher TG levels correlate with more severe pathological damage in mouse pancreatitis models [11]. This suggests that TG levels are associated with severe pancreatitis and pancreatic necrosis. The underlying mechanism may involve hypertriglyceridemia worsening pancreatic necrosis through the toxicity of free fatty acids [12]. During an AP episode, a large amount of pancreatic lipase is released into circulation, hydrolyzing serum TG and adipose tissue into excessive free fatty acids (FFAs). These FFAs damage pancreatic vascular endothelial cells and acinar cells, causing intracellular Ca^2+^ overload, inhibiting mitochondrial ATP production, and activating intracellular trypsin, ultimately leading to cellular necrosis [13]. Additionally, increased blood viscosity and accumulation of fatty acid complexes can obstruct pancreatic microvessels, exacerbating ischemia and acidosis [14]. Many clinical studies have confirmed the correlation between high TG levels and more severe pancreatitis [15]. Hypertriglyceridemia is an independent risk factor for early acute kidney injury and persistent organ failure in AP patients [16, 17]. Compared to AP of other etiologies, HTG-induced AP is associated with more severe outcomes, including higher rates of pancreatic necrosis, sepsis, and local complications [18].

Our results showed a statistically significant difference in Ca^2+^ levels between the MAP and MSAP/SAP groups (*P* = 0.002), with an inverse correlation to disease severity (B = −1.619). Hypocalcemia is a risk factor for moderate to severe HTG-AP (*P* = 0.013, OR = 0.167, 95% CI: 0.041–0.689) and demonstrates predictive value (AUC = 0.625, 95% CI: 0.55–0.7, *P* = 0.002). This finding aligns with previous clinical studies reporting significantly lower Ca^2+^ levels in severe pancreatitis compared to mild cases. Serum Ca^2+^ levels can predict AP severity, including in HTG-AP [3, 19, 20]. Ca^2+^ levels also predict acute kidney injury (AKI), organ failure, and endotoxemia in AP patients [21, 22]. During AP progression, large amounts of lipase hydrolyze adipose tissue into abundant FFAs, which chelate calcium ions in blood and tissues. These calcium ions deposit rapidly in fat necrosis areas, resulting in significant hypocalcemia. Additionally, increased glucagon release, insufficient parathyroid hormone response, altered calcium-albumin binding, and abnormal calcium distribution further affect Ca^2+^ levels. Intracellular Ca^2+^ is essential for maintaining normal pancreatic function. During HTG-AP, high unsaturated fatty acid concentrations cause sustained intracellular Ca^2+^ elevation in pancreatic acinar cells, inhibiting mitochondrial ATP production and promoting trypsin activation, ultimately causing cellular necrosis [23, 24]. Therefore, pharmacological agents targeting calcium signaling pathways, by inhibiting intracellular Ca^2+^ release, blocking extracellular Ca^2+^ influx, or enhancing Ca^2+^ clearance, have significant potential for future HTG-AP treatment [25–27].

CRP is an acute-phase protein produced by the liver. During infection, trauma, ischemia, or immune inflammation, cytokines released by immune cells stimulate significant CRP production. AP is an acute inflammatory condition caused by autodigestion of the pancreas. Results of this study indicate that elevated CRP levels represent a risk factor for moderate-to-severe pancreatitis. Numerous studies have confirmed that CRP levels reflect the inflammatory severity in AP, with higher CRP levels positively correlated with increased disease severity in HTG-AP patients. CRP is a valuable biomarker for predicting disease severity, complications, and mortality [3, 28, 29]. Studies have shown that CRP levels exceeding 150 mg/L within the first 72 h are closely related to pancreatic necrosis. Filipe S. Cardoso et al. found that CRP measured 48 h after admission provides superior predictive accuracy for severe pancreatitis, pancreatic necrosis, and in-hospital mortality compared to measurements at other times [30]. Recently, some studies have suggested that IL-6 may predict mortality and pancreatic necrosis more accurately than CRP in AP patients [31].

Our findings indicate that AMY is not an independent risk factor for moderate-to-severe HTG-AP. Amylase and lipase are indicators used to assess pancreatitis severity in animal studies. However, in clinical practice, AMY is primarily used as a diagnostic marker for AP rather than an indicator of disease severity [32]. Several case reports suggest that AMY levels may remain within normal ranges in patients with AP, chronic pancreatitis, hypertriglyceridemic pancreatitis, or advanced-stage pancreatitis [33, 34]. When pancreatic acinar cells are severely damaged, they may fail to release sufficient amounts of pancreatic enzymes, resulting in normal serum AMY levels [35]. Approximately half of patients with hypertriglyceridemic pancreatitis have AMY levels below three times the upper limit of normal [36]. A potential explanation is that plasma from hyperlipidemic patients may contain AMY activity inhibitors [37]. This characteristic complicates the clinical diagnosis of pancreatitis.

Apolipoprotein A (ApoA) is a major protein component of plasma lipoproteins and forms the structural and functional core of high-density lipoproteins (HDL). ApoA1 is its primary subtype. Its primary role involves initiating and facilitating reverse cholesterol transport—moving excess cholesterol from tissues to the liver for utilization or excretion. Additionally, ApoA1 exhibits antioxidant, anti-inflammatory, and antithrombotic effects. Hu et al. proposed that TG levels act only as an initiating factor in HTG-AP and do not necessarily correlate with disease severity. They suggested that ApoA < 0.705 µmol/L and ApoB < 0.495 µmol/L may predict disease severity [20]. Our results showed that ApoA1 levels were significantly lower in the MSAP/SAP group compared to the MAP group (*P* = 0.005). However, despite this significant difference, ApoA1 was not an independent risk factor for HTG-AP severity (*P* = 0.222) in binary logistic regression analysis. Possible explanations include high collinearity between ApoA1 and TG due to their close physiological relationship, with TG potentially being a more direct predictor. When combined in a predictive model, the contribution of ApoA1 may be overshadowed by TG and other inflammatory markers. The relatively small sample size may also contribute to this result. The predictive value of ApoA1 for HTG-AP severity requires further confirmation through larger and prospective studies.

Our study identified hypertriglyceridemia, hypocalcemia, elevated CRP, and elevated WBC as independent risk factors for moderately severe and severe HTG-AP. Although elevated WBC, CRP, and hypocalcemia are recognized severity markers for general AP, no specific models currently integrate these readily available parameters for bedside use in HTG-AP patients. Our predictive model addresses this clinical gap. The combined predictor integrating these biomarkers achieved an AUC of 0.837, sensitivity of 77.4%, and specificity of 75% at the optimal cutoff value, outperforming individual biomarkers alone. Bootstrap analysis demonstrated that 95% confidence intervals for regression coefficients of TG, Ca^2+^, CRP, and WBC excluded zero, confirming model stability. The Hosmer–Lemeshow goodness-of-fit test showed a *P*-value of 0.312 (> 0.05), indicating good model calibration. Thus, this predictive model may assist clinicians in early identification of high-risk HTG-AP patients.

This study has several limitations. First, the retrospective, single-center design may introduce selection and information biases. Second, due to incomplete data in the hospital’s electronic system, important unmeasured confounders (e.g., BMI, detailed alcohol intake, smoking history, genetic background) could not be fully assessed, potentially influencing model estimates. Third, although blood samples were taken before initiating treatment, variations in the time from symptom onset to hospital admission and sampling may have affected baseline biomarker levels. Finally, while the model underwent rigorous internal (bootstrap) validation and calibration assessment, the absence of independent external and temporal validation currently limits its widespread application. As an observational study, whether clinical decisions guided by this model ultimately improve patient outcomes requires further investigation.

This study proposes a promising but preliminary prediction tool, with ultimate clinical utility contingent on validation in prospective, multicenter cohorts. Future research could aim to: (1) Perform external and temporal validation through prospective multicenter studies to confirm the model’s generalizability; (2) Enhance and compare models by integrating early imaging findings, genetic markers, metabolic parameters, or employing machine-learning algorithms, followed by comparative studies with existing scoring systems; (3) Translate and clinically evaluate the model by incorporating it into decision-support systems and conducting randomized trials to assess whether model-guided early treatment reduces organ failure, shortens hospital stays, or improves survival outcomes, including health economic evaluations. Finally, as research advances in HTG-AP risk factors, targeted therapies addressing these factors, such as Ca^2+^ signaling modulators and novel lipid-lowering agents, may play an important role in future management strategies.

## Conclusions

Elevated TG, CRP, WBC, and decreased Ca^2+^ are independent risk factors for severe HTG-AP. A combined model using these readily available early parameters demonstrates strong predictive performance, stability, and calibration.

## Data Availability

The datasets generated and analyzed during the current study are available from the corresponding author on reasonable request.

## Abbreviations

AP: Acute Pancreatitis
HTG-AP: Hypertriglyceridemic Acute Pancreatitis
APACHE II: Acute Physiology and Chronic Health Evaluation Ⅱ
MCTSI: Modified CT Severity Index
MAP: Mild Acute Pancreatitis
MSAP: Moderately Severe Acute Pancreatitis
SAP: Severe Acute Pancreatitis
ROC: Receiver Operating Characteristic
TG: Triglyceride
WBC: White Blood Cell
CRP: C-reactive protein
AMY: Amylase
GLU: Blood Glucose
ApoA1: Apolipoprotein A1
Ca^2+^: Serum calcium
UREA: Urea nitrogen
Cre: Creatinine
ALT: Alanine Aminotransferase
AST: Aspartate Aminotransferase
ALP: Alkaline phosphatase
GGT: γ-glutamyltransferase
TBIL: Total bilirubin
SPSS: Statistical Package for the Social Sciences
IQR: Inter Quartile Ranges
AUC: Area Under the Curve
ICU: Intensive Care Unit
APFC: Acute Peripancreatic Fluid Collection
SIRS: Systemic Inflammatory Response Syndrome
ARF: Acute Renal Failure
ARDS: Acute Respiratory Distress Syndrome
IAH: Intra-Abdominal Hypertension
FFAs: Free Fatty Acids
ATP: Adenosine triphosphate
AKI: Acute Kidney Injury
ApoA: Apolipoprotein A
HDL: High-density Lipoproteins
